# Association Between Physical Fitness and Cardiovascular Health in Chilean Schoolchildren from the Metropolitan Region

**DOI:** 10.3390/nu17010182

**Published:** 2025-01-03

**Authors:** Fabian Vasquez, Gabriela Salazar, Sofia Vasquez, Jorge Torres

**Affiliations:** 1 School of Nutrition and Dietetics, Finis Terrae University, Providencia 7501014, Chile; 2 Institute of Nutrition and Food Technology, University of Chile, Santiago 8330111, Chile; 3 Faculty of Medicine, University of Chile, Santiago 8330111, Chile; sofia.vasquez.o@ug.uchile.cl; 4 Faculty of Health, Santo Tomas University, Talca 3460000, Chile; jorge.torres@inta.cl

**Keywords:** nutrient intake, physical fitness, cardiovascular health

## Abstract

**Background:** Cardiovascular diseases increasingly impact youth, with early development of risk factors such as obesity, hypertension, and inadequate nutrient intake. Proper nutrient intake and physical fitness are vital for reducing these risks, especially in pediatric populations. This study explores the connection between physical fitness, metabolic risk, and nutrient status among 1656 Chilean schoolchildren from diverse socio-economic backgrounds. **Methods:** Anthropometric measures included weight, height, skinfold thickness, waist circumference, and blood pressure. Physical fitness was assessed via handgrip strength, standing long jump, and a six-minute walk test. Nutrient intake was also evaluated, and a composite metabolic risk score was calculated based on waist circumference, skinfolds, and blood pressure. **Results:** Boys consistently outperformed girls in physical fitness tests, including grip strength and horizontal jump, with differences becoming more pronounced in higher grades and Tanner stages. Girls exhibited higher subcutaneous fat levels and obesity prevalence during later grades, highlighting gender-specific patterns in body composition. Better physical fitness was associated with lower waist circumference, skinfold thickness, and metabolic risk scores. A moderate correlation between aerobic fitness (distance/height) and blood pressure (r = 0.27, *p* = 0.01) was observed. Z-Score MR analysis revealed that students in the lowest fitness tertile exhibited significantly higher cardiovascular risk profiles compared to their fitter peers. **Conclusions:** Physical fitness plays a critical role in reducing cardiovascular risk in children. The findings underscore the importance of promoting gender- and age-specific interventions that include both aerobic and strength-based physical activities. Comprehensive school programs focusing on nutrition and physical activity are essential to mitigating cardiovascular risk and promoting long-term health outcomes. Future longitudinal studies are recommended to establish causal relationships and evaluate the impact of targeted interventions.

## 1. Introduction

Cardiovascular diseases (CVDs) are partly a pediatric problem, as the onset of atherosclerosis appears to occur in early childhood [1]. Atherosclerosis, characterized by the hardening and narrowing of the arteries due to the buildup of fatty plaques, is a pathological process that can begin to develop very early in life, often because of a combination of genetic and environmental factors. These factors include an unhealthy diet, lack of physical activity, and a genetic predisposition to hypertension and dyslipidemia.

The clustering of cardiovascular and metabolic risk factors, such as abdominal obesity, hypertension, insulin resistance, elevated triglycerides (TG), and low HDL cholesterol, has been observed in children and adolescents and tends to persist from childhood into adulthood [2,3]. This phenomenon of “clustering” of risk factors is particularly concerning because it increases the likelihood of developing CVD in later stages of life. The persistence of these risk factors underscores the need for early interventions to prevent the progression of cardiovascular disease. The clustering of these risk factors is considered an appropriate measure of cardiovascular health in young people, as composite risk scores can, to some extent, compensate for daily fluctuations in individual risk factors [4].

High cardiorespiratory fitness during childhood and adolescence has been associated with a healthier cardiovascular profile in these stages and into adulthood [5,6]. Cardiorespiratory fitness, often measured through tests such as the 20 m shuttle run test, reflects the heart and lungs’ ability to supply oxygen to the muscles during prolonged exercise. Numerous studies have shown that children and adolescents with high cardiorespiratory fitness are less likely to develop cardiovascular risk factors such as hypertension and dyslipidemia and have better overall metabolic health. Additionally, these benefits appear to persist into adulthood, highlighting the importance of promoting physical activity from an early age [7].

The role of muscular fitness has also been increasingly recognized in preventing chronic diseases, and the characteristics of metabolic syndrome have been negatively associated with muscular strength in both men and women [8,9]. Muscular fitness, which includes strength, endurance, and power, is crucial not only for physical performance but also for metabolic health. Research has shown that greater muscular strength is associated with lower body fat levels, better insulin sensitivity, and more favorable lipid profiles [10]. During childhood and adolescence, muscular fitness has been inversely associated with established and emerging cardiovascular risk factors [11,12]. This means that youth with better muscular fitness tend to have lower levels of risk factors such as obesity, hypertension, and dyslipidemia, potentially reducing their future risk of developing CVD.

However, there is limited knowledge about the independent association of muscular fitness with individual cardiovascular risk factors and clustered metabolic risk in youth [13]. Most studies have focused on cardiorespiratory fitness, leaving a gap in our understanding of the specific role of muscular fitness in the cardiovascular and metabolic health of young people. This research gap is concerning, as muscular fitness may offer unique benefits that are not fully captured by assessing only cardiorespiratory fitness.

The cross-sectional Healthy Lifestyle in Europe by Nutrition in Adolescence (HELENA-CSS) study offers the opportunity to expand the association between muscular fitness and metabolic risk observed in Norwegian youth to a broader population of European adolescents. This study is significant because it provides data from a large and diverse sample of adolescents from various European countries, allowing for a more comprehensive and generalizable assessment of these associations. The objective of the present study is to analyze the independent associations between muscular and cardiorespiratory fitness with clustered metabolic risk in schoolchildren and adolescents from 10 different European centers [14]. This analysis can provide valuable information for developing public health interventions aimed at improving fitness and reducing metabolic risk in young people.

In recent years, additional research has supported and expanded these findings. For example, García-Hermoso et al. [15] found that cardiorespiratory fitness and muscular strength are inversely associated with the risk of metabolic syndrome in children and adolescents. This study reinforces the idea that both types of fitness play a crucial role in metabolic health. Another study by Kim et al. [16] showed that adolescents with higher levels of muscular strength had more favorable cardiovascular risk profiles, even after adjusting for cardiorespiratory fitness. Additionally, Ortega et al. [17] demonstrated that the combination of high cardiorespiratory and muscular fitness provides the greatest benefits for cardiovascular and metabolic health in adolescents. This finding underscores the importance of incorporating both aerobic and strength training exercises into physical activity recommendations for young people. In more recent research, García-Hermoso et al. [18] indicated that exercise-based interventions combining aerobic and resistance activities are effective in improving metabolic risk factors in overweight and obese adolescents. Finally, a study by Aadland et al. [19] emphasized the importance of regular physical activity and fitness in preventing obesity and improving metabolic health in children and adolescents. These recent studies reinforce and expand the existing evidence, highlighting the importance of physical fitness for cardiovascular and metabolic health in youth.

The study hypothesis was that schoolchildren with lower levels of physical fitness present greater metabolic risks, reflected in parameters such as body mass index (BMI), waist circumference, skinfold thickness, and blood pressure.

The objective was to analyze the association between physical condition and metabolic risk in schoolchildren from the metropolitan region of Chile, evaluating how different indicators of physical condition (such as grip strength and horizontal jump) are related to metabolic risk parameters (including waist circumference, body mass index, and blood pressure).

## 2. Materials and Methods

### 2.1. Study Population

In total, 1656 schoolchildren from first to eighth grade from public school, subsidized private school, and private schools in the communes of Cerro Navia, Macul, Providencia, Pedro Aguirre Cerda, Pudahuel, Quinta Normal, Santiago, San Miguel, and Vitacura participated in this study. The sample size calculation was based on previous studies on associations between physical fitness and metabolic risk in similar pediatric populations [20] Janssen & LeBlanc, 2010). The calculation considered a statistical power of 80% and a significance level of 5%, which allows for the detection of associations of moderate magnitude between fitness indicators and metabolic risk factors in this population. Stratified sampling was used based on the characteristics of the metropolitan region of Chile. The distribution of school types (public, private subsidized, and private) was made according to the proportions found in the urban population, allowing for an equitable representation of the different socio-economic contexts and cultural diversity of the region. The percentage distribution was similar to the proportion found in the urban Metropolitan Region (14% private, 61% subsidized private, and 25% public). The sample was evenly distributed in terms of age and gender. Each grade, from first to eighth, included a balanced number of students, with nearly 50% of each gender represented at every grade level. This uniform distribution ensures the representativeness of the sample and minimizes potential biases associated with age or gender differences.

Inclusion and exclusion criteria were applied as follows: the study included only schoolchildren without chronic health conditions that could affect the results in physical and cardiovascular health parameters. This ensured that the data obtained accurately represented the general student population. The additional exclusion criteria were non-attendance on the day of the measurements, conditions that limited the performance of the physical tests or cases where informed consent or assent was not provided.

Ethical approval was obtained from the INTA Ethics Committee of the University of Chile, and informed consent was provided by all participants and their legal guardians. Participants were selected across grades and balanced for gender, and all participants were Chilean with no ethnic differences.

### 2.2. Methodology

#### Anthropometry

Two nutritionists using standardized procedures performed the measurements. The intra-observer technical error of measurement and mean observer bias were within the limits suggested by the World Health Organization (WHO) [21]. Anthropometry methods were based on those used in the third U.S. National Health and Nutrition Examination Survey [21]. Each anthropometric measurement, including weight, height, waist circumference, and skinfold thickness, was taken in duplicate. A third measurement was conducted if the difference between the first two exceeded 0.5 cm for height and waist circumference or 1 mm for skinfold thickness. The average of the two closest values was recorded to ensure accuracy and reliability of the data.

1. Weight and height: Weight was obtained with the schoolchild standing at the center of the scale (SECA Ltd., Hamburg, Germany) wearing light clothing (no shoes, long pants, sweaters, or shirts) and without support. Height measurement was taken with heels together, arms along the body, and back against the scale (SECA Ltd.), with the head in the Frankfurt plane. The BMI Z-Score was calculated based on the 2007 WHO reference for sex and age to classify nutritional status [22]. Studies in Latin America and Chile have shown that these references are consistent with the distribution of BMI in local populations. This reference has been previously validated in Latin American populations with similar characteristics and is widely accepted in the region.
2. Skinfold thickness (Total body fat): Skinfolds (triceps, biceps, suprailiac, and subscapular) were measured with a LANGE caliper [23]. The mid-upper arm circumference was measured at the midpoint between the acromion and olecranon, with the arm extended and relaxed, without depressing the skin. The sum of skinfolds was used as a measure of subcutaneous fat [24]. The intra-observer technical error of measurement and mean observer bias were within the limits suggested by the World Health Organization (WHO) [21].
3. Waist circumference: A fiberglass tape measure was used to measure waist circumference at the top of the iliac crest, ensuring that the tape did not compress and was parallel to the floor. The measurement was taken at the end of a normal expiration [25].
4. Blood pressure: A sphygmomanometer was used to measure blood pressure after the schoolchild had been seated for 10 min, with the arm supported and at heart level. The NANHES 2004 standard [26] and the 90th percentile were used to determine the proportion of schoolchildren with altered blood pressure (systolic or diastolic) [27].
5. Puberty: Pubertal development was classified using Tanner stages based on breast development in girls and genital development in boys [28]. Pubertal status was assessed by a nutritionist who received specialized training in using the Tanner scale prior to the study. The training included theoretical sessions on the stages of pubertal development and practical workshops supervised by a pediatric endocrinologist. The nutritionist was evaluated for accuracy and consistency during a pilot phase to ensure reliability in identifying the stages of pubertal development. This rigorous preparation ensured that the assessments were conducted with precision and adherence to clinical standards.

### 2.3. Physical Fitness Tests

1. Handgrip dynamometry: Handgrip strength (kg) was measured with a digital dynamometer (TKK 5101 Grip-D; Takei, Tokyo, Japan). The size of the right hand was measured to find the optimal grip span [29].
2. Standing long jump: To measure lower body explosive strength, the schoolchild stood behind the jump line with feet shoulder-width apart. They bent their knees and swung their arms before jumping as far as possible [30].
3. Six-minute walk test: This functional cardiorespiratory test measures the maximum distance covered in six min. It was performed on a flat, non-slip surface with a minimum perimeter of 30 m [31].
4. Heart rate: Heart rate was measured with a POLAR heart rate monitor before, during, and after the walk test. It was recorded in three consecutive periods: (a) Pre-test, after three minutes in a seated position; (b) Minute by minute during the test; (c) Post-exercise recovery in each of the three minutes following the test [32].

Physical fitness indicators are less common in pediatric studies; however, they allow muscle strength to be accurately evaluated [18]. This study is the first of its kind to analyze the association between physical fitness and cardiovascular health in Chilean schoolchildren from the metropolitan region, filling a gap in local research and underlining the importance of these health indicators in this specific population.

### 2.4. Metabolic Risk Factors

A continuous score representing a composite metabolic risk factor profile was computed from the following variables: waist circumference, sum of four skinfolds, systolic blood pressure, and diastolic blood pressure. Each of these variables was standardized as follows: standardized value = (value − mean)/SD, separately for boys and girls and by 1 yr age groups. The Z-Scores of the individual risk factors were summed to create the metabolic risk score (Z-Score MR). A lower metabolic risk score is indicative of a better overall CVD risk factor profile [33].

### 2.5. Statistics

The Kolmogorov−Smirnov test was used to assess the normality of distributions, and Levene’s test was applied to confirm homogeneity of variances in-group comparisons (e.g., between genders or types of establishments). These checks ensure that the analyses meet the required statistical assumptions, increasing the reliability of the results. The Chi-Square test (χ^2^) was used to determine significant differences when comparing groups of observed frequencies with expected frequencies. The Kruskal−Wallis test was used to determine significant differences when comparing non-normal samples of a quantitative variable in three groups, and the T-Test was used for the comparison of normal samples. For the handling of missing data, mean imputation was used for those cases where the missing data were less than 5%, ensuring the validity of the analyses without introducing significant bias. In the case of missing data greater than 5%, they were discarded from the corresponding analysis to avoid distortions. Analyses were performed using STATA version 18.0 and *p* < 0.05 determined statistical significance (StataCorp. 2023. Stata Statistical Software: Release 18. StataCorp LLC (College Station, TX, USA)).

## 3. Results

Table 1 presents the distribution of participants by grade and gender, including both the number and percentage of students in each category. The sample consisted of 1656 participants, evenly distributed between males and females, with 828 (50.00%) participants of each gender. The data are broken down across eight grades, from first to eighth, showing that the proportion of males and females in each grade is nearly equal, with variations remaining within a range of 1.28 percentage points.

For example, in first grade, there were 109 male participants (49.6%) and 111 female participants (50.5%), contributing to 220 students in that grade. Similarly, in eighth grade, there were 98 males (49.0%) and 102 females (51.0%), making 200 participants. This consistent balance in gender distribution across all grades underscores the representativeness of the sample.

The table highlights the uniformity of the sample distribution by gender and grade, ensuring that the findings can be interpreted reliably without significant gender or grade-related biases. This balance supports the generalizability of the study’s conclusions to similar populations.

Table 2 reveals significant differences in anthropometric and physical fitness variables across grades, Tanner stages, and genders. Weight and height showed a progressive increase with advancing grades and Tanner stages, with boys consistently exhibiting higher values than girls, particularly in the later stages of development. Girls, however, had a higher sum of skinfolds across most grades, reflecting greater subcutaneous fat accumulation, a difference that became more pronounced during advanced Tanner stages. Interestingly, obesity prevalence was higher among boys in earlier grades but either equalized or was surpassed by girls in intermediate and later grades.

Waist circumference above the 90th percentile showed a slightly higher prevalence among boys in early grades, whereas girls demonstrated higher rates in intermediate and later grades. Blood pressure above the 90th percentile remained consistently low across grades but displayed gender differences, with boys being more affected during Tanner stages 4 and 5. These findings suggest that gender-specific patterns in anthropometric health metrics become increasingly evident as children progress through puberty.

In terms of physical fitness, boys consistently outperformed girls in measures such as horizontal jump and grip strength, with notable improvements seen in later Tanner stages. Boys also exhibited superior endurance, achieving longer distances in the six-minute test, with peak performance during Tanner stage 5. Statistical analyses confirmed significant gender differences in weight, height, horizontal jump, and grip strength (*p* < 0.05), emphasizing the critical influence of Tanner stage and grade on physical development. 

The results in Table 3 show significant correlations between physical fitness tests, adjusted for height or weight, and cardiovascular health variables in schoolchildren. A strong negative correlation was found between waist circumference and both distance/height (r = −0.45, *p* = 0.001) and sum of skinfolds (r = −0.38, *p* = 0.001), indicating that better performance in these fitness tests is associated with smaller waist circumferences and lower body fat. Similarly, there was a strong positive correlation between grip strength/weight and both waist circumference (r = 0.56, *p* = 0.0001) and sum of skinfolds (r = −0.48, *p* = 0.001), suggesting that higher muscular strength is linked to improved body composition.

Additionally, a significant positive correlation was found between distance/height and blood pressure (r = 0.27, *p* = 0.01), implying that better aerobic fitness is moderately associated with lower blood pressure. No significant correlations were found for jump/height with blood pressure.

Table 4 presents the Z-Score MR cut-off points for different combinations of physical tests, adjusted for sex and developmental stage according to Tanner. The table shows the associations between Distance/Height and two additional tests: Jump/Height and Grip Strength/Weight, allowing us to observe how cardiovascular risk values vary according to performance in each test combination. In the first section of the table, the Z-Score MR cut-off points for the Low, Medium, and High categories of Distance/Height combined with the Jump/Height levels are shown. In this combination, schoolchildren with high performances in both tests (High category in Distance/Height and Jump/Height) have the lowest Z-Score MR values, indicating a lower cardiovascular risk profile (Z-Score MR = −1.03). In contrast, those with low performance in both tests show elevated Z-Score MR values, suggesting a higher cardiovascular risk (Z-Score MR = 1.86). The second section of the table presents Z-Score MR cut-off points for Distance/Height in combination with Grip Strength/Weight. Similar to the first combination, students with high performance in both tests show lower Z-Score MR values (Z-Score MR = −1.10), while those with low performance in both tests record the highest Z-Score MR values (Z-Score MR = 2.10), indicating a less favorable cardiovascular risk profile.

**Figure 1:**
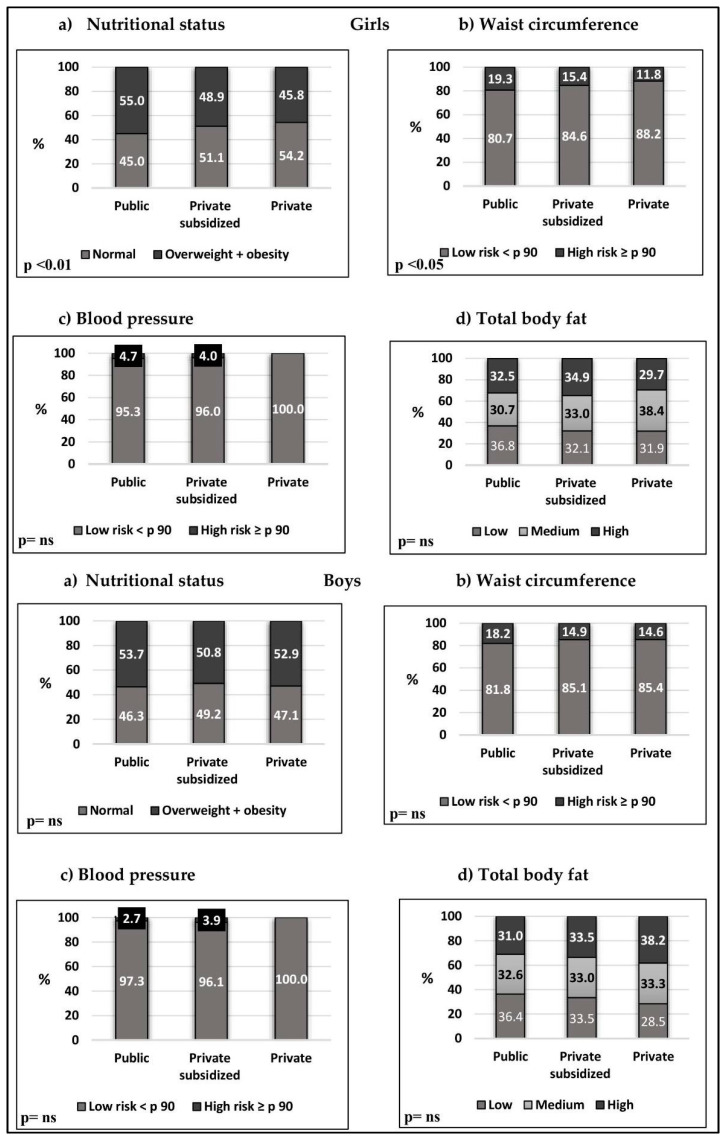
Nutritional status indicators by tertiles in boys and girls according to educational establishment.

Figure 1 shows the comparison, by type of school, of nutritional status, waist circumference, blood pressure and total body fat in boys and girls, respectively.

Girls from public schools show a significantly higher proportion of malnutrition due to excess weight and waist circumference compared to girls from subsidized and private schools (*p* < 0.05). No significant differences were detected in blood pressure and total body fat variables. In boys, no significant differences were observed across types of educational establishments.

**Figure 2:**
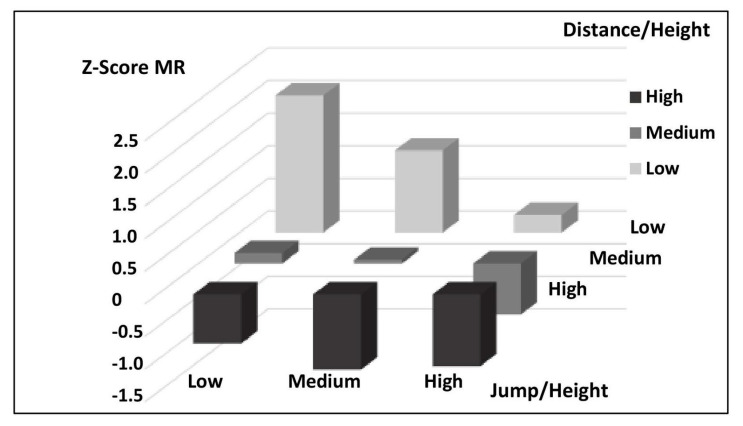
Z-Score MR vs. high, medium, and low tertiles of horizontal jump (m) and distance covered in the walk test (m) adjusted for gender, grade, and Tanner stage.

Figure 2 illustrates the relationship between Z-Score MR and the tertiles of performance in the horizontal jump and distance covered in the walk test. Schoolchildren in the high tertile for both tests show lower Z-Scores, indicating better cardiovascular health, while those in the low tertile demonstrate higher Z-Scores, suggesting higher cardiovascular risk.

**Figure 3:**
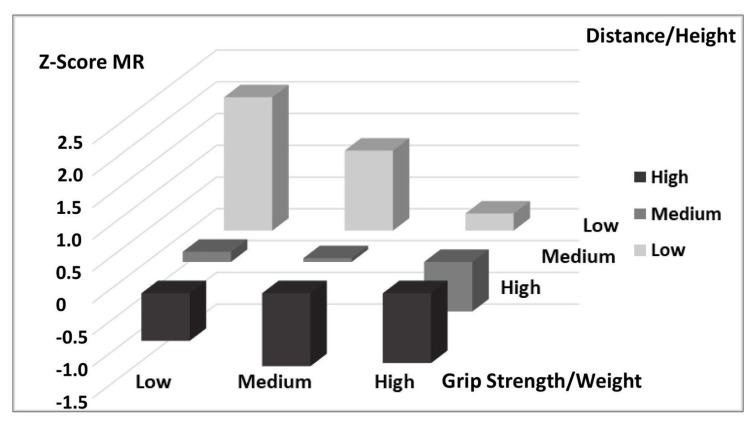
Z-Score MR vs. high, medium, and low tertiles of distance (m) and grip strength (kg) adjusted for gender, grade, and Tanner stage.

Figure 3 shows the Z-Score MR in relation to the tertiles of performance in distance covered and grip strength. As in Figure 3, those in the high tertile for both tests display lower Z-Scores, indicating better cardiovascular health outcomes, whereas those in the low tertile exhibit higher Z-Scores, reflecting increased cardiovascular risk.

## 4. Discussion

The relationship between nutrition, physical activity, and cardiovascular health in schoolchildren is a critical area of research given the global increase in childhood obesity rates and cardiovascular diseases [34]. This study examines differences in anthropometric indicators, physical fitness, and cardiovascular risk in schoolchildren from different grades and genders. The findings highlight significant disparities in obesity and physical fitness that can influence these children’s future health.

This study highlights the complex interplay between nutrition, physical activity, and cardiovascular health in schoolchildren, providing critical insights into anthropometric indicators, physical fitness, and cardiovascular risk. The results demonstrate a progressive increase in weight and height as children advance in grade and Tanner stage, consistent with expected growth patterns. Boys exhibited higher values for these parameters, particularly in later Tanner stages, while girls showed greater subcutaneous fat accumulation, reflected in consistently higher sum of skinfolds. Obesity prevalence was higher among boys in earlier grades but was surpassed by girls in later grades, underscoring the importance of gender-specific approaches to managing weight and associated risks during puberty [20,35]. Childhood obesity is a known predictor of cardiovascular diseases in adulthood [36], and early interventions are crucial to mitigate this risk.

Waist circumference is an important indicator of central adiposity and cardiovascular risk [37]. In this study, waist circumference > p90 and blood pressure > p90, critical indicators of cardiovascular risk, revealed significant gender differences. Boys exhibited higher prevalence of waist circumference > p90 in early grades, while girls showed higher rates in later grades. Prevalence of blood pressure > p90 remained relatively low but demonstrated a trend of higher rates in boys during Tanner stages 4 and 5. Waist circumference and blood pressure are well-established indicators of central adiposity and cardiovascular risk, emphasizing the need for targeted interventions to address these metrics in schoolchildren [38,39,40,41].

The physical fitness measures provided further insights into these disparities. Boys consistently outperformed girls in horizontal jump, grip strength, and the six-minute distance test, with differences becoming more pronounced in higher grades and Tanner stages. These trends reflect the influence of pubertal development on muscular and aerobic capacities. Correlations between physical fitness and cardiovascular health indicators highlight the protective effects of fitness; for instance, better performance in distance/height was associated with lower waist circumference and skinfolds, while a moderate correlation with blood pressure suggests external factors may confound this relationship [10,33,42]. Schoolchildren with elevated waist circumference also showed a higher prevalence of elevated blood pressure, a significant risk factor for cardiovascular diseases [43]. The association between obesity and high blood pressure in children has been well documented [44], and our results underscore the need to monitor these parameters in school health programs.

These findings coincide with studies showing that children with better physical fitness have a lower risk of developing metabolic risk factors [45]. Regular physical activity is crucial for maintaining good cardiovascular health, and our results suggest that physical education programs should be a priority in all schools, especially those with fewer resources.

Subcutaneous body fat was higher in girls and schoolchildren in the lowest fitness tertile, which is concerning given that higher adiposity is associated with poorer metabolic health [46]. The difference in body fat between sexes could be explained by hormonal and developmental factors [47], but it also underscores the need for gender-specific interventions. Physical performance was lower in schoolchildren from public establishments, suggesting possible differences in opportunities for physical activity or the quality of physical education provided. Promoting a school environment that encourages regular physical activity is essential for children’s physical development and long-term health [48].

The observed correlations between physical fitness and cardiovascular health variables are consistent with the existing literature. For example, waist circumference showed a strong correlation with grip strength, reflecting the interrelationship between central adiposity and muscular strength [49]. The sum of skinfolds also negatively correlated with physical performance, which is consistent with studies indicating that excessive adiposity can limit physical capacity [50]. Systolic blood pressure, although less prevalent, still showed an association with physical fitness. Studies have shown that physical activity can improve blood pressure in children [51], and our results support the inclusion of regular physical activities to manage blood pressure and promote cardiovascular health. The correlation observed between the distance/height variable and blood pressure is statistically significant but weak. This relationship suggests that, although there is an association between the level of aerobic fitness and blood pressure, its impact on clinical practice may be limited. This type of weak correlation may not reflect a strong causal relationship, but rather a partial association that could be influenced by other factors not controlled for in the study. It is important to consider that blood pressure in schoolchildren can be affected by multiple external factors, such as stress, family environment, eating habits, and hours of sleep, among others [44,52]. These factors, which were not controlled for in this study, could have influenced blood pressure variability and, therefore, the observed correlation with physical condition. Furthermore, natural variability in blood pressure among children, influenced by growth and development, could reduce the strength of any observed associations with fitness variables in a sample of school-age children [43]. The use of the Z-Score MR provides a useful tool for assessing cardiovascular risk in schoolchildren. Schoolchildren in the lowest tertile of physical fitness tests had a significantly higher Z-Score MR, indicating a higher risk of cardiovascular diseases. These results are consistent with studies showing that low physical fitness is associated with poorer cardiovascular risk profiles [4]. The importance of muscular strength and aerobic capacity in cardiovascular health is well documented. Evidence suggests that even moderate levels of physical activity can have significant health benefits [20]. Therefore, promoting regular physical activity at all ages is crucial to reducing the risk of cardiovascular diseases in the future.

The results of this study have important public health implications. The disparity in obesity and physical fitness among different types of educational establishments suggests the need for policies that ensure equal opportunities for physical activity and nutritional education. Specific programs targeted at low-resource schoolchildren may be necessary to address these disparities [53]. Additionally, implementing physical activity programs that include both aerobic and strength training exercises can be an effective strategy to improve schoolchildren’s cardiovascular health. Integrating physical activities into the school curriculum and promoting healthy habits from an early age are essential to prevent chronic diseases and improve long-term health [54].

The sample in this study is representative of the school population in the metropolitan region of Chile, including students from first through eighth grade across various types of educational institutions (public, subsidized, and private). The sample was balanced in terms of both gender and grade level, with approximately 50% of participants being male and 50% female in each grade. This uniformity in age and gender distribution strengthens the external validity of our findings, allowing generalization to school populations with similar sociodemographic characteristics. Additionally, the stratified sampling approach ensures that the data accurately reflect the socioeconomic diversity of the region. By appropriately representing gender, grade levels, and types of institutions, the study results provide a reliable overview of physical fitness and metabolic risk factors in the school population. This representativeness minimizes potential biases and enhances the applicability of our conclusions for informing school health policies and intervention programs aimed at improving cardiovascular health among students.

This study highlights the importance of nutrition and physical activity in the cardiovascular health of schoolchildren. Significant differences in obesity and physical fitness among different types of educational establishments underscore the need for specific interventions to reduce health disparities. Promoting regular physical activity and a balanced diet is crucial for physical development and the prevention of cardiovascular diseases in childhood and adulthood. 

Limitations and future directions: Limitations of the study include the lack of a longitudinal evaluation to analyze the long-term impact of physical activity on cardiovascular health. Future studies should explore specific interventions for each age group and the role of physical education in the school curriculum.

The cross-sectional nature of the study limits the ability to establish causal relationships. Therefore, future longitudinal studies are necessary to establish causal relationships. The observed results reflect associations, and not direct causal effects, between physical condition and metabolic risk [43,52]. Furthermore, it is emphasized that uncontrolled external factors, such as stress levels, sleep hours, and the family environment, which could affect both blood pressure and physical performance in schoolchildren [44,52], can influence the variability in the results. In addition, it is suggested to investigate how factors such as stress and the family environment influence the development of physical condition and cardiovascular risk in the Chilean school population to observe the effects of changes in lifestyle on cardiovascular risk factors in this population. 

## 5. Conclusions

This study provides critical insights into the anthropometric, physical fitness, and cardiovascular health profiles of schoolchildren, emphasizing the importance of puberty as a pivotal period for the development of health disparities. The findings demonstrate gender- and grade-specific trends in key indicators, including weight, height, sum of skinfolds, obesity prevalence, waist circumference, and blood pressure. Boys consistently exhibited higher weight and height, while girls showed greater subcutaneous fat accumulation. Notably, obesity prevalence shifted from being higher in boys during early grades to being higher in girls in later grades, underscoring the dynamic nature of these risk factors during pubertal development.

The inclusion of physical fitness measures, such as horizontal jump, grip strength, and six-minute walk distance, provided valuable insights into their protective effects on cardiovascular health. Boys outperformed girls in these measures, with differences becoming more pronounced in higher grades and Tanner stages. The strong correlations between physical fitness and cardiovascular health indicators, including lower waist circumference and skinfolds, highlight the importance of fitness in mitigating cardiovascular risks. However, the moderate correlation between aerobic fitness and blood pressure suggests that external factors, such as stress, sleep patterns, and environmental influences, may also play a role.

The use of the Z-Score MR offered a novel perspective for assessing cardiovascular risk, revealing that better physical fitness profiles were associated with significantly lower cardiovascular risk. These findings reinforce the critical role of promoting regular physical activity and balanced nutrition during childhood and adolescence to improve long-term health outcomes and reduce the risk of chronic diseases.

Future longitudinal studies are necessary to establish causal relationships and explore the impact of tailored interventions. Addressing uncontrolled external factors and understanding their interplay with physical fitness and metabolic risk are essential for developing effective public health strategies. These results provide a foundation for advancing policies and interventions aimed at fostering healthier school environments and improving cardiovascular health in children.

## Figures and Tables

**Table 1:** Distribution of participants by grade and gender.

Grade	Male n (%)	Female n (%)	Total n (%)
1	109 (49.6)	111 (50.5)	220
2	109 (50.5)	107 (49.5)	216
3	107 (49.1)	111 (50.9)	218
4	96 (50.0)	96 (50.0)	192
5	100 (51.3)	95 (48.7)	195
6	106 (50.2)	105 (49.8)	211
7	103 (50.5)	101 (49.5)	204
8	98 (49.0)	102 (51.0)	200
Total	828 (50.0)	828 (50.0)	1656 (100.0)

**Table 2:** Anthropometric, physical fitness, and health indicators by grade and gender.

	Grades							
Variable	1 (n = 220)	2 (n = 216)	3 (n = 218)	4 (n = 192)	5 (n = 195)	6 (n = 211)	7 (n = 204)	8 (n = 200)
Boys (n = 828)								
Weight (k)	27.1 ± 5.1	29.6 ± 6.7	33.7 ± 9.5	36.8 ± 8.0	42.0 ± 9.6	47.1 ± 10.7	55.9 ± 11.8 *	57.6 ± 14.9
Height (m)	123.5 ± 5.7 *	128.0 ± 6.5	132.5 ± 6.3	138.9 ± 6.5	144.4 ± 7.9	150.7 ± 8.0	158.8 ± 7.9 *	161.9 ± 11.1 *
Sum of skinfolds (mm)	59.1 ± 6.7	61.2 ± 7.7	65.0 ± 9.8	66.2 ± 8.2	68.9 ± 8.4	72.1 ± 9.2	77.1 ± 10.2 *	76.5 ± 11.6
Obesity (%)	22	27.5	28.9	24.0 **	21.0	17.9	19.4	16.3
Waist circumference > p90 (%)	16.5	14.5	23.5	19.8	13.0	10.4 **	18.5	17.4
Blood pressure > p90 (%)	1.2	2.6	2.5	2.1	3.0	1.9	3.9	12.2
Horizontal jump (m)	1.0 ± 0.2 *	1.1 ± 0.2 *	1.2 ± 0.2 *	1.2 ± 0.2 *	1.3 ± 0.2 *	1.4 ± 0.2 *	1.4 ± 0.2 *	1.6 ± 0.3 *
Grip strength (k)	10.6 ± 2.0	12.1 ± 2.7	13.5 ± 2.3	16.1 ± 2.7 *	17.8 ± 3.8	20.9 ± 4.9	25.7 ± 6.3 *	30.4 ± 7.5 *
Distance 6-min test (m)	575.5 ± 55.9	618.3 ± 49.5	643.3 ± 54.8	672.1 ± 46.9 *	680.1 ± 60.1	686.9 ± 53.4 *	683.3 ± 55.5 *	715.6 ± 61.7 *
Girls (n = 828)								
Weight (k)	25.8 ± 4.9	29.5 ± 4.8	34.1 ± 7.0	36.5 ± 7.8	42.0 ± 9.0	47.6 ± 11.4	52.2 ± 10.3 *	56.2 ± 11.5
Height (m)	121.1 ± 5.9 *	127.6 ± 4.7	133.8 ± 5.7	137.9 ± 6.9	144.1 ± 7.8	148.9 ± 6.3	154.9 ± 5.9 *	157.4 ± 6.2 *
Sum of skinfolds (mm)	58.4 ± 6.6	61.5 ± 6.8	64.6 ± 8.1	65.6 ± 8.5	68.3 ± 8.6	72.7 ± 9.6	72.9 ± 9.6 *	75.7 ± 9.9
Obesity (%)	21.6	17.0	23.4	12.5 **	12.6	20.9	11.0	14.7
Waist circumference > p90 (%)	13.3	14.7	22.5	17.7	17.7	22.9 **	22.9	14.7
Blood pressure > p90 (%)	1.0	1.3	4.5	4.2	1.0	1.9	1.9	8.8
Horizontal jump (m)	0.9 ± 0.1 *	1.0 ± 0.2 *	1.0 ± 0.2 *	1.1 ± 0.2 *	1.2 ± 0.2 *	1.2 ± 0.2 *	1.2 ± 0.2 *	1.2 ± 0.2 *
Grip strength (k)	10.1 ± 0.8	11.6 ± 2.0	13.2 ± 2.5	15.2 ± 2.9 *	17.5 ± 3.8	20.0 ± 3.9	21.7 ± 3.8 *	23.6 ± 3.8 *
Distance 6-min test (m)	578.7 ± 51.1	617.1 ± 46.1	639.4 ± 45.7	654.6 ± 45.7 *	671.3 ± 45.2	659.4 ± 51.6 *	657.7 ± 49.8 *	657.8 ± 49.4 *

* Student’s *t*-test *p* < 0.05. ** Proportions test *p* < 0.05.

**Table 3:** Correlations (r) between physical fitness tests and variables related to cardiovascular health (adjusted for gender, grade, and Tanner stage).

Variable	Distance/Height	Jump/Height	Grip Strength/Weight
Waist circumference	−0.45	0.13	0.56
*p*	0.001	0.0001	0.0001
Sum of skinfolds	−0.38	−0.43	−0.48
*p*	0.001	0.001	0.001
Blood pressure	0.27	---	---
*p*	0.01		

**Table 4:** Cut-off points for Z-Score MR by categories of Distance/Height combined with Jump/Height and Grip Strength/Weight tests (adjusted for gender, grade, and Tanner stage).

Distance/Height	Jump/Height (Z-Score MR)			Grip Strength/Weight (Z-Score MR)		
	Low	Medium	High	Low	Medium	High
High	−0.58	−0.79	−1.03	−0.75	−1.15	−1.10
Medium	0.36	0.23	−0.47	0.16	0.06	−0.78
Low	1.86	0.89	−0.17	2.10	1.26	0.27

## Data Availability

The original contributions presented in this study are included in the article. Further inquiries can be directed to the corresponding author(s).
